# Acute intermittent porphyria: a disease with low penetrance and high heterogeneity

**DOI:** 10.3389/fgene.2024.1374965

**Published:** 2024-08-12

**Authors:** Jia-Jia Lei, Shuang Li, Bai-Xue Dong, Jing Yang, Yi Ren

**Affiliations:** ^1^ Department of First Clinical Medical School, Shanxi Medical University, Taiyuan, China; ^2^ Department of Endocrinology, The First Hospital of Shanxi Medical University, Taiyuan, China

**Keywords:** acute intermittent porphyria, hydroxymethylbilane synthase, gene mutation, penetrance, heterogeneity, oligogenic inheritance

## Abstract

Acute intermittent porphyria (AIP) is caused by mutations in the gene encoding hydroxymethylbilane synthase (HMBS), a key enzyme in the heme biosynthesis pathway. AIP is an autosomal dominant disorder characterized by low penetrance and a highly heterogenous clinical presentation. The estimated prevalence of AIP is 5–10 cases per 100,000 persons, with acute attacks manifesting in less than 1% of the at-risk population. This low frequency of attacks suggests significant roles for oligogenic inheritance and environmental factors in the pathogenesis of the disease. In recent years, identification of several modifier genes has advanced our understanding of the factors influencing AIP penetrance and disease severity. This review summarizes these factors including the impact of specific *HMBS* mutations, oligogenic inheritance, mitochondrial DNA copy number, age, sex, the influence of sex hormones, and the role of environmental factors. Further studies into the etiology of AIP disease penetrance should inform pathogenesis, potentially allowing for the development of more precise diagnostic and therapeutic approaches.

## 1 Introduction

The porphyrias are a group of disorders of porphyrin metabolism caused by defective enzyme activity in one of the eight enzymes of the heme biosynthesis pathway. These are primarily hereditary disorders, and divided into eight subtypes (Kauppinen, 2005). According to the tissue in which the particular enzyme defect is located, porphyrias are classified as hepatic porphyrias, caused by disorders of porphyrin metabolism in the liver, and erythropoietic porphyrias, disorders of porphyrin metabolism in the bone marrow. Hepatic porphyrias have also been subdivided into acute hepatic porphyrias (AHPs) and chronic hepatic porphyrias according to the characteristics of the disease attacks.

Acute intermittent porphyria (AIP), the most common form of acute hepatic porphyria, is an autosomal dominant disorder caused by a partial deficiency in the activity of the third enzyme in the heme biosynthesis pathway, hydroxymethylbilane synthase (HMBS), also known as porphobilinogen deaminase (PBGD) (Puy et al., 2010). AIP rarely attacks before puberty, with typical symptoms occurring between the ages of 20 and 40, more in females than males. In patients with AIP, certain endogenous or exogenous factors such as fluctuations in female sex hormones, low caloric intake, alcohol consumption, stress, infections, and porphyrinogenic drugs lead to the upregulation of hepatic δ-aminolevulinic acid synthase 1 (ALAS1). This results in the accumulation of the porphyrin precursors δ-aminolevulinic acid (ALA) and porphobilinogen (PBG) in AIP patients, ultimately causing the development of clinical symptoms (Anderson et al., 2005; Schulenburg-Brand et al., 2022).

Patients with pathogenic *HMBS* can be categorized into those with manifest AIP (MAIP) and those with latent AIP (LAIP) (Barreda-Sánchez et al., 2019). A patient with LAIP is someone who has never experienced acute porphyria-related manifestations (Sardh and Barbaro, 1993). Patients with MAIP usually present with acute neurovisceral attacks, including severe abdominal pain, nausea and vomiting, constipation, tachycardia, hypertension, psychiatric disorders (e.g., insomnia, anxiety, and depression), and severe neurologic symptoms (neuropathy and seizures) (Puy et al., 2010; Souza et al., 2021).

AIP management includes avoidance of triggers, oral or intravenous glucose and intravenous hemin infusion, symptom treatment, acute attack prevention, and management of long-term complications (Schulenburg-Brand et al., 2022; Wang et al., 2023). Intravenous glucose (300 g/d in adults) is used in early acute attacks or when hemin is unavailable, but hemin infusion is more effective (Schulenburg-Brand et al., 2022; Wang et al., 2023). Hemin infusion rapidly downregulates ALAS1 expression, decreases ALA and PBG accumulation, and resolves symptoms in 48–72 h, although its effectiveness in preventing recurrent attacks is unclear (Yarra et al., 2019; Blaylock et al., 2020). Lists of safe and unsafe drugs for patients with AIP are available online (http://www.drugs-porphyria.org/; https://porphyrianet.org/).

Givosiran (Givlaari), an ALAS1-directed siRNA, is approved for treating adults with AHP in the United States and those 12 years and older in the EU (Balwani et al., 2020; Scott, 2020). Monthly givosiran injections significantly reduced the rate of acute attacks in a phase 3 study in patients with recurrent AIP (Balwani et al., 2020). A Phase III clinical trial of Givosiran in AHP patients showed sustained improvements in clinical manifestations and quality of life with a favorable safety profile (Kuter et al., 2023). Thus, patients with recurrent acute attacks (4 or more per year) should be considered for prophylactic treatment with heme therapy or subcutaneous givosiran (Wang et al., 2023). Liver transplantation may be considered for patients with persistent severe disease who have failed other treatment options. In addition, gene therapy, hPBGD mRNA, and pharmacological chaperone therapy are promising treatments for AIP (Bustad et al., 2020; Kizilaslan et al., 2023).

In addition to variable penetrance, there is considerable heterogeneity in disease severity, even within the same family. Most symptomatic AIP patients have only a few attacks in their lifetime; however, about 3%–8% of symptomatic AIP patients have recurrent attacks (Gouya et al., 2020). The variation in penetrance and clinical phenotype would indicate that other genetic and/or environmental factors are either preventing or precipitating AIP attacks in *HMBS* heterozygotes (outlined in Table 1).

**TABLE 1:** Genetic factors in the heterogeneity of acute intermittent porphyria.

	Subjects	Research indicators	Indicator differences	Possible mechanisms	References
Complex mutations in the *HMBS* gene (NC_000011.10)					
Different mutation sites have different phenotypes	333 AIP patients in Finland	Penetrance of acute attacks at different mutation sites of AIP	Relatively severe: A penetrance of >50% in mutations c.593G>A, c.76C>T, c.1073delA	Pathogenicity varies across mutation sites	Baumann and Kauppinen (2020)
			Relatively mild: A penetrance of ≤20% in mutations c.499C>T, c.673C>G, c.33G>T, c.517C>T		
	468 AIP patients in northern Sweden	Severity of clinical manifestations/penetrance	Severe: p. Trp198X (44%) p. Arg173Trp (50%) Mild: p. Arg167Trp (13%)	HMBS residual enzyme activity levels vary. p. Trp198X (low or zero p. Arg173Trp (2%) p. Arg167Trp (5%)	Andersson et al. (2000)
	AIP patients (602 manifest patients, 1,968 relatives) from French reference center for porphyria and the general population from exome variant server (EVS; 12,990 alleles)	Penetrance	AIP families/general population: (22.9%/0.5%–1%)	AIP susceptibility is regulated by the inheritance of mutations in the HMBS gene, other genetic factors, and environmental factors	Lenglet et al. (2018)
		The frequencies of null alleles and missense mutations in AIP families/general population	AIP families/general population: null allele (74%/0%) missense mutations (26%/100%) Null alleles are associated with more severe phenotypes and higher penetrance than missense mutant alleles		
	117 *HMBS* gene mutations from reported AIP patients	Software prediction: effect of *HMBS* mutations on the structure and function of the corresponding proteins	Severe: p. Arg116Trp p. Arg173Trp, p. Arg149X, p. Gln217His, p. Gly218Arg, p. Ala219Pro, p. Ala330Pro	R149, Q217, G218, and A219 interact directly with reaction intermediates and DPM cofactors Residue A330 has no direct interaction with the reaction intermediates, but non-synonymous mutations on it disrupt the secondary structure of the protein and affect the other five residues	Fu et al. (2019)
			Relatively moderate: p. Arg26Cys p. Asp99Asn, p. Arg167Trp, p. Gly168X, p. Gln194X, p. Gly221Asp	These amino acids are relatively far from the active site and interact directly with reaction intermediates and DPM cofactors	
			Mild: p. Thr35Met p. Gly111Arg, p. Leu238Pro	These amino acids are far from the active site and have no direct interaction with reaction intermediates and DPM cofactors	
Potential oligogenic inheritance					
*CYP2D6*	50 Spanish AIP genetic carriers. (most of these patients carried the founder mutation c.669-698del30	CYP2D6*4/*5 allelic frequencies	CYP2D6*4/*5: Total AIP (0.12/0.01) LAIP (0.19/0.02) MAIP (0.06/0) MAIP was less frequent in defective CYP2D6*4 and *5 allele carriers	Defective *CYP2D6* alleles: i) May be less sensitive to porphyrogenic xenobiotic intermediate metabolites ii) May consume less heme than normal alleles	Barreda-Sánchez et al. (2019)
*PEPT2*	122 French AIP patients	Severity of renal failure	PEPT2*1 carriers (more severe chronic renal failure)	PEPT2*1 has a high affinity for ALA, and ALA is reabsorbed more efficiently by renal tubules	Tchernitchko et al. (2017)
	*PEPT2* deficient mice	Severity of neurotoxicity	PEPT2*2 carriers (more significant neurotoxicity)	PEPT2*2 has low affinity for ALA and low ALA efflux in the brain	Hu et al. (2007)
Multiple genes in the nervous system	153 Russian AIP patients and 302 of their relatives	Neurological clinical manifestations	*AGRN*, *DOK7*, and *SCN4A* genes were associated with congenital myasthenic syndromes (CMS), the symptoms of which are significantly similar to the neurological features of AIP attacks	The *HMBS* gene mutation combined with defects in neurological regulatory genes may play a key role in triggering acute AIP attacks and developing specific symptom profiles	Goncharova et al. (2019)
*PPARA*	Pathogenic variants of AIP	Protein function	*PPARA* gene and ALAS1 have obvious overlap in function	Mutations in the *PPARA* gene may affect the onset of AIP by regulating the CYP450 system, thus affecting the biosynthetic pathway of heme	Fu et al. (2019) Thomas et al. (2015)
Mitochondrial DNA					
Human peripheral blood leukocytes	34 Italian AIP patients and 37 healthy people	Mitochondrial DNA copy number	All AIP patients had the low number of mitochondria (more pronounced in symptomatic patients)	Energy metabolism disorders	Di Pierro et al. (2023)
		Penetrance	The higher mitochondrial DNA content is associated with decreased porphyria severity or even incomplete disease penetrance		
Human serum	34 Italian AIP patients and 37 healthy people	Serum levels of PERM1	PERM1 levels were significantly lower in patients with AIP, and this impairment was more pronounced in symptomatic patients		

## 2 Genetic factors

### 2.1 Complexity of mutations in the *HMBS* gene

*HMBS* is the gene responsible for AIP, with mutations resulting in loss of enzyme function. The *HMBS* gene is located on chromosome 11 (11q24.1–24.2), and the coding region consists of 15 exons. Transcription from different promoters generates two types of transcripts, the housekeeping transcripts and the erythroid-specific transcripts, and this article focuses on the housekeeping transcript (NM_000190.4). To date, more than 500 different *HMBS* pathogenic mutations have been reported in the Human Gene Mutation Database (Stenson et al., 2017). *HMBS* mutations include missense mutations, nonsense mutations, splice mutations, frameshift mutations, large/small deletions, large/small insertions, and large/small duplications. There are 31 CpG dinucleotides in the *HMBS* 1,086 base pair coding sequence (Chen et al., 2016). The primary sources of DNA sequence variation are replication errors and deamidation of CpG dinucleotides, which are hotspots for mutations (Cooper and Youssoufian, 1988). Most AIP mutations are found only in single or a few unrelated families, demonstrating the molecular genetic heterogeneity of AIP (Whatley et al., 1999; Martinez di Montemuros et al., 2000; Ulbrichova et al., 2009; Chen et al., 2019a). However, there exist some relatively common mutations: one reason is due to CpG dinucleotide mutation hotspots in the gene (Tjensvoll et al., 2003), such as the mutations encoding p.Arg173Trp, p.Arg167 Gln, and p.Arg116Trp; and the second reason is the founder effect, such as *HMBS* c.593G>A (p.Trp198X) in Sweden and Norway (Tjensvoll et al., 2003), c.669_698del30 in the Murcia region of Spain (Guillén-Navarro et al., 2004).

Although correlation between AIP genotypes and phenotypes has not been established (Fraunberg et al., 2005; Bustad et al., 2013; Bonkovsky et al., 2014), studies suggest that different mutations can lead to different phenotypes and differences in clinical penetrance (Chen et al., 2016; Zhang et al., 2021). For instance, the c.1073delA, c.593G>A (p. Trp198X), and c.76C>T (p. Arg26Cys) mutations have been associated with higher penetrance (greater than 50%) or a more severe clinical presentation (Andersson et al., 2000; Fraunberg et al., 2005; Baumann and Kauppinen, 2020). Conversely, the c.33G>T, c.499C>T (p. Arg167Trp), and c.673C>G (p. Arg225Gly) mutations have been associated with lower penetrance (less than or equal to 20%) or milder phenotypes (Andersson et al., 2000; Fraunberg et al., 2005; Baumann and Kauppinen, 2020). However, in northern Sweden, the p. Arg173Trp mutation had a higher clinical penetrance compared to Finland (Andersson et al., 2000; Baumann and Kauppinen, 2020). Recently, a study of protein function prediction (Fu et al., 2019) showed that some mutations, including p. Arg149X, p. Gln217His, p. Gly218Arg, p. Ala219Pro, and p. Ala330Pro, lead to severe clinical manifestations. Moreover, concordant with a previous study, it was also shown that patients with p. Arg116Trp and p. Arg173Trp mutations had severe clinical symptoms (Bustad et al., 2013). In contrast, the p. Arg26Cys, p. Asp99Asn, p. Arg167Trp, p. Gly168X, p. Gln194X, and p. Gly221Asp mutations result in relatively moderate disease while mutations p. Thr35Met, p. Gly111Arg, and p. Leu238Pro exhibited a mild clinical phenotype. The penetrance of AIP in different Finland families varied even though the genotypes were the same (Baumann and Kauppinen, 2020), again suggesting that modifier genes and environmental factors also affect AIP phenotype.

### 2.2 Potential oligogenic inheritance model

A study of AIP in France using patients from the French Reference Center for Porphyria and sequencing data derived from the general population evaluated the prevalence and penetrance of AIP, and the influence of genetic factors on the phenotypic expression of *HMBS* mutations (Lenglet et al., 2018). Among the patients and their families of the French Reference Center for Porphyria, 496 patients with MAIP and 1,672 LAIP relatives were identified, and the penetrance of AIP in these AIP families was estimated to be 22.9%. The Exome Variant Server (EVS) database was then used to estimate the prevalence of deleterious *HMBS* mutations and AIP penetrance in the general population. A total of 42 missense mutations were identified in the AIP family (n = 20), and the EVS database (n = 22), and their pathogenicity was assessed by computerized and functional studies, which revealed a minimum estimated prevalence of AIP of 1/1,299 in the general population. Thus, given the estimated prevalence of pathogenic *HMBS* mutations in France, between 50,000 and 100,000 AIP patients (rather than 496) would be expected, suggesting the penetrance of *HMBS* mutations in the general population is only 0.5%–1%. This is in keeping with a study using whole exome sequencing of more than 40,000 Caucasians indicating that the AIP penetrance in *HMBS* heterozygotes is ∼1% (Chen et al., 2016).

Further analysis of intra-familial correlations for AIP revealed that correlations were generally strong and moderated by the individual’s kinship and region of residence. Disease penetrance in affected families was estimated to range from 58% (parent-offspring) to 73% (siblings).

Finally, Lenglet et al. analyzed the effects of the specific mutations, and then assessed the frequency of missense mutations and null alleles in the EVS database (general population) (n = 6,495) and in families of AIP patients, including simplex families (n = 106), multiplex families (n = 147), and patients with recurrent AIP attacks (4 or more per year; n = 37). Notably, these different mutations were associated with variable clinical phenotypes. Null alleles (little or no expression of HMBS) were more frequent in multiplex families than in simplex families, and patients with recurrent AIP also had a higher frequency of null genes than less severe forms, but the difference was not statistically significant. In the general population, the null allele frequency was 0%, and the frequency of missense mutations 100%. In familial AIP patients, the null allele frequency was 74%, and missense mutations 26%, suggesting that null alleles are associated with more severe phenotypes and higher penetrance than other mutant alleles.

These researchers proposed an oligogenic genetic model with environmental modifiers to better explain the penetrance and heritability of AIP (Lenglet et al., 2018).

#### 2.2.1 Protective variants

##### 2.2.1.1 *CYP2D6* gene

Recent research has indicated a higher frequency of specific cytochrome P450 (*CYP*) alleles among individuals with certain types of porphyria compared to the general population, suggesting a potential role of these alleles in disease susceptibility (Christiansen et al., 2000; Gardlo et al., 2003). An investigative study conducted in Argentina focused on analyzing *CYP2D6* polymorphisms in patients with porphyria, encompassing 121 participants, including 51 healthy volunteers, 50 porphyria patients, and 19 individuals with elevated iron levels. The results showed that the CYP2D6*3 and CYP2D6*4 alleles, particularly, were linked to the onset of porphyria (Lavandera et al., 2006). Furthermore, a recent study in Spain investigated the correlation between hepatic *CYP* genes and acute attacks of AIP (Barreda-Sánchez et al., 2019). The study examined CYP2C9*2, *3, CYP2C19*2, CYP2D*4, *5, CYP3A4*1B, and CYP3A5*3 alleles in fifty Spanish carriers of the AIP gene. Most fifty patients with AIP (78%) carried the founder mutation c.669_698del30. The Spanish study suggested that *CYP* genes may be penetrance modifiers in AIP because CYP2D6*4 and *5 are more frequent in LAIP than MAIP, despite the similar allele frequencies of CYP2D6*4 and *5 in the total AIP carriers and the general population (Menoyo et al., 2006), suggests CYP2D6*4 and *5 defective alleles play a protective role in the clinical onset of AIP, modulating the penetrance of AIP.

#### 2.2.2 Damaging variants

##### 2.2.2.1 *PEPT2* gene

Human peptide transporter protein 2 (PEPT2) is expressed in proximal renal tubular cells, and its genetic variation modulates the severity of porphyrin-related nephropathy and neurological impairment (Yasuda et al., 2019). ALA, a highly hydrophilic compound, is freely filtered in the urine and is reabsorbed by human peptide transporter protein 2 (PEPT2) (Tchernitchko et al., 2017). It has been demonstrated that the toxicity of porphyrin metabolites is associated with the pathogenesis of typical renal tubulointerstitial lesions (Pallet et al., 2015). During AIP attacks, ALA accumulates and promotes renal tubular cell death and tubulointerstitial damage. ALA and PBG promote phenotypic changes and apoptosis in renal epithelial cells, and an epithelial-to-mesenchymal transition that leads to primary tubulointerstitial nephropathy.

Recently, a study showed that in French Caucasians with *HMBS* mutations, *PEPT2* variants affect the severity and prognosis of porphyria-associated nephropathy (Tchernitchko et al., 2017). Haplotype analysis demonstrated the existence of two significant variants of *PEPT2*, PEPT2*1 and PEPT2*2. PEPT2*1 has a high affinity for ALA, and PEPT2*2 has a low affinity for ALA. Individuals carrying the high-affinity transporter (PEPT2*1) may suffer from more severe chronic renal failure because ALA is reabsorbed more efficiently by the renal tubules and is, therefore, more nephrotoxic.

The development of neuropsychiatric symptoms in AIP may be associated with an increase in brain ALA (Pischik and Kauppinen, 2009; Fu et al., 2019). It has been found that the protein encoded by the *PEPT2* gene is responsible for the transport of ALA from the cerebrospinal fluid (Hu et al., 2007). Since PEPT2*2 has a lower affinity for ALA than PEPT2*1, more significant neurotoxicity may occur in PEPT2*2 carriers because they have lower ALA efflux from the brain (Ennis et al., 2003; Jaramillo-Calle et al., 2019).

##### 2.2.2.2 Multiple genes in the nervous system

During acute attacks of AIP, the toxic porphyrin precursors ALA and PBG accumulate in tissues, causing neurologic injury. Recently, a study from Russia performed long-term follow-up and genetic analysis of 153 patients with AIP and 302 of their relatives, focusing on the *HMBS* gene mutation spectrum and the degree of penetrance (Goncharova et al., 2019). In whole-exome sequencing of six AIP patients the researchers found several pathogenic gene variants associated with neurodegenerative diseases, including *UNC13A*, *ALG8*, *FBXO38*, *AGRN*, *DOK7*, and *SCN4A*. *AGRN*, *DOK7*, and *SCN4A* genes were associated with congenital myasthenic syndromes (CMS), the symptoms of which are significantly similar to the neurological features of AIP attacks. Thus, *HMBS* gene mutation combined with defects in neurological regulatory genes may play a key role in triggering acute AIP attacks and developing specific symptom profiles (Goncharova et al., 2019). However, this hypothesis requires confirmatory studies.

##### 2.2.2.3 *PPARA* gene

Peroxisome proliferator-activated receptor (PPARα) is a ligand-activated transcription factor and member of the nuclear hormone receptor superfamily. A recent study on pathogenic variants of AIP showed a significant functional overlap of the *PPARA* gene with ALAS1 by protein functional analysis (Fu et al., 2019).

Most drugs are metabolized by the cytochrome p450 system. Studies have shown that some drugs induce ALAS1 either directly or indirectly by inducing synthesis of these heme-containing CYP450 proteins (Granick, 1963; Granick, 1966), leading to the overproduction of heme precursors (Podvinec et al., 2004; Bissell et al., 2017). A study showed that *PPARA* genes directly regulate transcription of *CYP2C8* and *CYP3A4* (Thomas et al., 2013; Thomas et al., 2015), consistent with findings that the *PPARA* gene can specifically regulate *CYP1-3* class of genes (Richert et al., 2003; Barbier et al., 2004; Sérée et al., 2004). Consequently, mutations in *PPARA* may influence AIP attacks by modulating the CYP450 system, thus impacting the heme biosynthesis pathway (Thomas et al., 2015). Despite these findings, the importance of *PPARα* in expression of the AIP phenotype remains unexplored.

### 2.3 Mitochondrial DNA

Mitochondria are essential organelles in all nucleated cells, involved in cellular respiration, metabolism, macromolecular biosynthesis, and regulation of apoptosis, cell proliferation, and motility. Mutations, deletions, and insertions in mitochondrial DNA play a crucial role in the pathogenesis of inherited mitochondrial diseases.

Liver transcriptome analysis in an animal model of AIP showed dysregulation of genes associated with mitochondrial biogenesis (Chen et al., 2019b), suggesting that a lack of functionally normal mitochondria may lead to (or be a toxic effect of) acute attacks of AIP. In addition, abnormalities in mitochondrial morphology and function have been reported in studies of animal models of AIP (Homedan et al., 2014; Homedan et al., 2015) and patients with porphyria (Ostrowski et al., 1983; Dixon et al., 2019), emphasizing the potential role of impaired energy supply in the clinical manifestations of the disease.

Recently, an Italian study reported a link between decreased mitochondrial DNA content and porphyria (Di Pierro et al., 2023). The study performed a relative quantification of mitochondrial DNA (mtDNA) copy number in human peripheral leukocytes to indirectly estimate the average mitochondrial content per cell in patients with symptomatic and asymptomatic AIP and compared these results with those of healthy controls. The results showed that AIP patients had decreased numbers of mitochondria and significantly lower serum PERM1 (a marker of mitochondrial biogenesis) levels; a reduction more pronounced in symptomatic patients, suggesting that higher mitochondrial DNA content may modulate porphyria severity or penetrance, perhaps through affecting an individual’s ability to respond to endogenous or environmental stressors.

If confirmed in further studies, mitochondrial DNA copy number per cell and/or mitochondrial biogenesis may become biomarkers of porphyria. The metabolic and genetic factors responsible for these results remain to be determined.

## 3 Gender, age, sex hormones

The mutation that causes AIP is autosomal and inherited equally between men and women (Schuurmans et al., 2001; Kauppinen and von und zu Fraunberg, 2002). However, acute attacks affect females more often than males, and recurrent attacks are more common in females (Chen et al., 2016).

Most AIP onset attacks occur between the ages of 20 and 40, with onset in childhood rare (Varshney et al., 2018). In addition to a three-year-old Nigerian boy diagnosed with AIP (Sykes, 2001), a Chinese study has reported childhood AIP onset with two female cases and one male case, ages 1 year and 7 months, 1 year and 5 months and 5 years old (mutations: c.579_583del, c.499C > T, c.422 + 1G > A, respectively) (Ren et al., 2023).

A recent Finland study (Baumann and Kauppinen, 2020) found that in families with AIP, the penetrance of hospitalized attacks in female patients was as high as 41%, and the penetrance of all acute symptoms associated with AIP was 50%. Similar findings have been reported in families with the founder mutation p. Trp198X in northern Sweden (Andersson et al., 2000). In addition, a study in Cape Town, South Africa, showed a male-to-female ratio of 1:6 for acute attacks in patients with AIP and a high correlation between AIP attacks and menstruation (Hift and Meissner, 2005). Another Finland study evaluating the prognosis of 206 adult patients with porphyria found that nearly one-third of the women had symptoms of porphyria associated with their menstrual cycle (Kauppinen and Mustajoki, 1992), while a study in northern Sweden showed that symptoms in AIP patients change during the menstrual cycle (Ahangari et al., 2015).

The above studies suggest that female hormones trigger AIP attacks, which may explain the higher penetrance in female patients with AIP than in male patients.

Hift and Meissner (2005) reported that the effect of sex hormones is more pronounced in AIP than in other types of porphyria, with 50% of female AIP attacks associated with the menstrual cycle (De Block et al., 1999; Innala et al., 2010; Linenberger and Fertrin, 2020), and 10%–30% of these attacks occurring during the luteal phase (Ahangari et al., 2015).

Progesterone and its metabolites induce the hepatic ALAS1 enzyme (Podvinec et al., 2004; Phillips, 2019) and are associated with increased heme metabolism (Bonkovsky et al., 2019; Spiritos et al., 2019), and may be a more potent inducer than estrogen (Andersson et al., 2002). While estrogen has a weak effect on AIP, when combined with progesterone, it can induce porphyria attacks (Bissell et al., 2017). Monitoring serum progesterone levels can clarify the luteal phase and progesterone-induced attacks in patients with AIP who experience acute attacks.

Gonadotropin-releasing hormone (GnRH) analogs are synthetic hypothalamic hormone analogs that include gonadotropin-releasing hormone agonists (GnRH-a) and gonadotropin-releasing hormone antagonists (GnRH-A). Studies have shown that GnRH-a therapy inhibits endogenous hormone production and successfully prevents attacks in patients with AIP associated with the menstrual cycle (Castelo-Branco et al., 2001; Innala et al., 2010; Xu et al., 2022). However, the administration of GnRH-a has an initial rebound effect of transiently stimulating the pituitary gland to secrete elevated Follicle-stimulating Hormone (FSH) and Luteinising Hormone (LH), known as the “flare-up” effect, which leads to a transient increase in ovarian hormone production that lasts for about 7 days. This flare-up effect promotes the maturation of primordial follicles and improves therapeutic efficacy but may increase the risk of ovarian hyperstimulation syndrome (OHSS) (Lambalk et al., 2017). GnRH-A inhibits ovarian function by directly inhibiting GnRH, which decreases the levels of FSH and LH secreted by the pituitary gland without any flare-up effect and has a more rapid onset of action.

Elagolix is an oral GnRH-A that is FDA-approved for the treatment of moderate to severe pain associated with endometriosis (Lamb, 2018), however unlike leuprolide, it induces *CYP3A* and a fluorescence-based drug porphyrogenicity screening assay in Leghorn Male Hepatoma (LMH) cells demonstrated that it is porphyrogenic and associated with increased acute porphyria attacks in women (Ma and Bonkovsky, 2022).

In addition, animal model studies have found an increased local vasoconstrictor response in AIP mice and a lower sensitivity to vasodilation in female mice (pro-constrictor response), suggesting the sex difference may be due to differing sensitivities of mesenteric arteries to porphyrin precursors (Pulgar et al., 2019).

## 4 Environmental factors

AIP attacks can be triggered by low energy intake, dieting, and alcohol consumption (Storjord et al., 2019). Alcohol, as a porphyrinogenic agent, enhances the induction of ALAS1, causing acute AIP attacks (Goldberg et al., 1981; Doss et al., 2000). Peroxisome proliferator-activated receptor γ coactivator 1α (PGC-1α) is an enzyme that regulates ALAS1 in the liver and can be activated by fasting (Herzig et al., 2001). Fasting can cause a significant increase in PGC-1α and ALAS1 (Scassa et al., 1998), which may lead to AIP attacks. Smoking may contribute to recurrent AIP attacks by influencing drug metabolism and steroid hormone metabolism (Michnovicz et al., 1986; Klaiber and Broverman, 1988; Lip et al., 1991).

The activity of ALAS1 is positively regulated by substances that induce hepatic *CYPs* (Manceau et al., 2017). As discussed in Section 2.2.2.3, most porphyrogenic drugs cause AIP attacks through impacting CYP450 metabolism, particularly through induction of, or irreversible inactivation of, *CYP3A4* and *CYP2C9* (Brodie, 1980; Moore and Hift, 1997; Thunell, 2000; Thunell et al., 2007). Studies have shown that phenobarbital is extremely porphyrinogenic and induces drug-oxidizing enzymes in the liver, exacerbating AIP (Kappas et al., 1977; Holroyd and Seward, 1999). Phenobarbital-induced AIP mice have upregulated *CYP2C40*, *CYP2C68*, and *CYP2C69*, equivalent to human *CYP2C8*, *CYP2C9*, and *CYP2C19* respectively (Nelson et al., 2004; Zanger et al., 2008), but downregulates *CYP21A1* (Chen et al., 2019b), equivalent to human *CYP21A2* or a homolog of *CYP17A1* (−37), a crucial enzyme in corticosteroid and sex hormone synthesis (Idkowiak et al., 1993). Diclofenac and fluoxetine, *CYP3A4* inhibitors, are associated with acute porphyrias (Pelkonen et al., 2008). *CYP2C8* and *CYP3A4/5* metabolize hydroxychloroquine *in vivo* (Rendic and Guengerich, 2020). AIP attacks have been reported during hydroxychloroquine treatment for systemic lupus erythematosus (Esteve-Valverde et al., 2020), and with use of the HIV antiretroviral drugs, atazanavir/ritonavir (Le Tiec et al., 2005; Bharti et al., 2016).

Heme is a potent inhibitor of ALAS1 expression and activity. Studies have shown that heme oxygenase (HO-1) is induced by stress, hypoglycemia, toxins, heavy metals, and organic chemicals (Thunell, 2000; Li et al., 2007; Abraham and Kappas, 2008), leading to increased heme catabolism and a decrease in the intracellular heme pool, which activates ALAS1 transcription. Chronic administration of enflurane or isoflurane and treatment with griseofulvin have been shown to induce an increase in HO-1 activity. Chronic exposure to enflurane and isoflurane anesthesia, as well as veronal treatment, increased HO mRNA expression (Rodriguez et al., 2005; Ferrándiz and Devesa, 2008). Furthermore, a mouse model of AIP has confirmed the porphyrinogenicity of isoflurane and sevoflurane (Ruspini et al., 2018).

## 5 Concluding remarks and future perspectives

In conclusion, it is important to consider the low penetrance of AIP and highly variable clinical phenotype, as revealed in population studies. This variability may result in underdiagnosis of the disease, or undertreatment, or even be potentially life-threatening for some patients. A comprehensive understanding of the prevalence and penetrance of this rare disease aids early diagnosis, the establishment of standardized management protocols, and identification of potential therapeutic targets. We have described analyses of genetic susceptibility factors and modifier genes implicated in the regulation of AIP penetrance and phenotype, however, most of these findings require further studies to validate their clinical relevance. Nonetheless the characterization of confounding variables holds promise for enhancing the clinical care of AIP patients and may pave the way for the development of novel, safe, and productive treatment strategies.
